# Design and Analysis of Bar-seq Experiments

**DOI:** 10.1534/g3.113.008565

**Published:** 2013-11-05

**Authors:** David G. Robinson, Wei Chen, John D. Storey, David Gresham

**Affiliations:** * Lewis-Sigler Institute for Integrative Genomics, Princeton University, Princeton, New Jersey 08544; † Berlin Institute for Medical Systems Biology, Max-Delbrück-Center for Molecular Medicine, 13125 Berlin, Germany; ‡ Center for Genomics and Systems Biology, Department of Biology, New York University, New York, New York 10003

**Keywords:** yeast, Bar-seq, galactose, functional genomics, *Sacchromyces cerevisiae*

## Abstract

High-throughput quantitative DNA sequencing enables the parallel phenotyping of pools of thousands of mutants. However, the appropriate analytical methods and experimental design that maximize the efficiency of these methods while maintaining statistical power are currently unknown. Here, we have used Bar-seq analysis of the *Saccharomyces cerevisiae* yeast deletion library to systematically test the effect of experimental design parameters and sequence read depth on experimental results. We present computational methods that efficiently and accurately estimate effect sizes and their statistical significance by adapting existing methods for RNA-seq analysis. Using simulated variation of experimental designs, we found that biological replicates are critical for statistical analysis of Bar-seq data, whereas technical replicates are of less value. By subsampling sequence reads, we found that when using four-fold biological replication, 6 million reads per condition achieved 96% power to detect a two-fold change (or more) at a 5% false discovery rate. Our guidelines for experimental design and computational analysis enables the study of the yeast deletion collection in up to 30 different conditions in a single sequencing lane. These findings are relevant to a variety of pooled genetic screening methods that use high-throughput quantitative DNA sequencing, including Tn-seq.

Uncovering the connection between genotype and phenotype remains one of the central challenges of modern genetics. At the same time, the rate at which new genomes are sequenced currently outpaces our capacity to functionally annotate those genomes. Addressing these challenges requires efficient means of quantifying phenotypes associated with defined genetic perturbations. Methods for uniquely identifying and quantifying phenotypic effects of mutant alleles in complex mixtures enable the parallel analysis of hundreds to thousands of genotypes. Pooled mutant analysis entails the use of either libraries of defined mutants tagged with unique DNA sequences (molecular barcodes) (Winzeler *et al.* 1999; Giaever *et al.* 2002) or complex libraries of tens of thousands of unique mutants generated by random insertional mutagenesis. Analogously, comprehensive libraries of short hairpin RNAs (shRNAs) enable parallel analysis of perturbations of mammalian genes in cell culture (Schlabach *et al.* 2008; Silva *et al.* 2008; Sims *et al.* 2011).

Recently, methods for estimating mutant abundances in complex mixtures have been introduced that capitalize on advances in high-throughput quantitative DNA sequencing. Barcode analysis by sequencing (Bar-seq) was first developed to analyze libraries of thousands of *Saccharomyces cerevisiae* gene deletion mutants (Smith *et al.* 2009) and has subsequently been used to analyze a library of deletion mutants in *Schizzosaccharomyces pombe* (Han *et al.* 2010). The use of Bar-seq enables efficient, accurate, and comprehensive genetic screens for addressing a variety of questions, such as defining the genetic requirements for initiation and maintenance of cell quiescence in response to distinct starvation signals (Gresham *et al.* 2011). In organisms for which barcoded mutant libraries are not available, high-throughput DNA sequencing of pools of transposon insertion mutants (Tn-seq) enables multiplexed mutant analysis. Tn-seq was initially applied in studies of *Streptococcus pneumonia* (van Opijnen *et al.* 2009) and *Haemophilus influenzae* (Gawronski *et al.* 2009) and has subsequently been adapted for use in diverse organisms (Brutinel and Gralnick 2012; Gallagher *et al.* 2011). Similarly, PhiTSeq facilitates simultaneous analysis of thousands of transposon-mutagenized haploid human cells (Carette *et al.* 2011). The widespread adoption of pooled mutant screens using high-throughput quantitative DNA sequencing attests to the power of these methods for efficient genetic analysis.

In contrast to the rapid technological advances in pooled mutant analysis, there has not yet been a statistical treatment of the experimental design and analysis of data generated by high-throughput DNA sequence analysis of these complex libraries. Thus, major methodological and analytical questions remain unanswered. What is the appropriate statistical framework for analyzing DNA sequence count data? What are the sources of variation? What is the appropriate study design for maximizing the power and accuracy to detect differences in mutant abundances? What sequence read depth maximizes the precision of these methods while minimizing the cost and resources required?

We undertook a study that aimed to address these questions with the goal of providing guidance for the design and analysis of pooled mutant screens using high-throughput DNA sequencing. Using experimental analysis of the *S. cerevisiae* gene deletion collection in two different conditions, we studied the contribution of treatment and biological and technical variation to Bar-seq data (Figure 1). We demonstrated that the negative binomial models used to analyze RNA-seq data are also directly applicable to Bar-seq data. Using computational subsampling of our experimental data, we studied the effect of different experimental designs on the results from Bar-seq analysis. We found that biological replicates substantially improved statistical power, whereas technical replicates provided only moderate additional statistical power. We also found that increasing sequencing depth beyond 6 million reads per condition provided limited improvement in the experimental results, regardless of experimental design.

**Figure 1:**
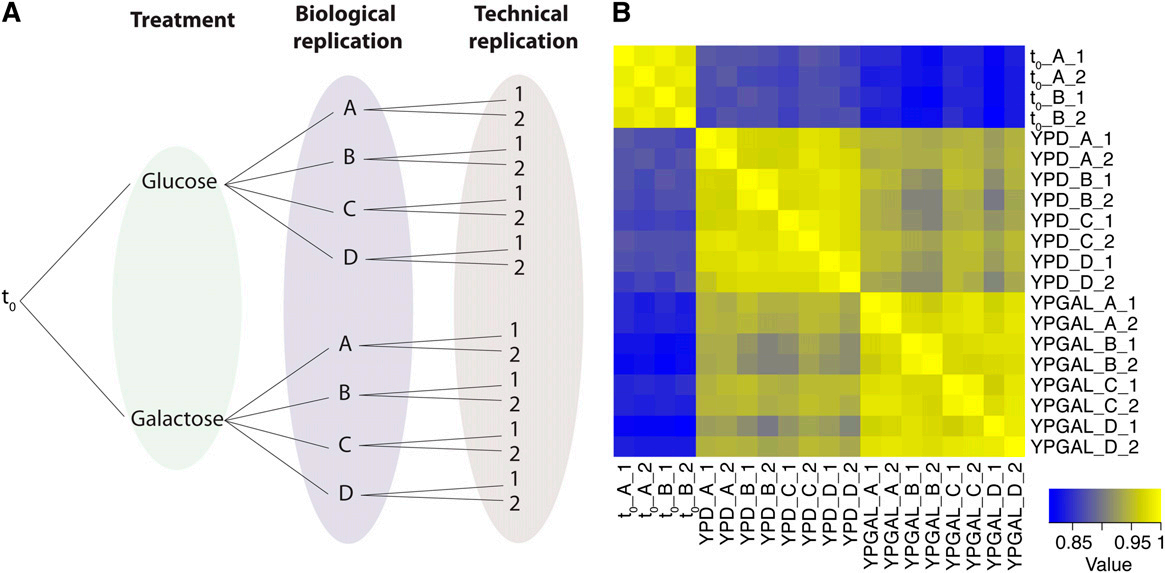
Experimental design and results. (A) Our experimental design entailed two treatments (twenty-four hours of growth in glucose/YPD or galactose/YPGal), four biological replicates, and two technical replicates, along with four samples at time point 0 [not shown in (A)]. (B) Heat map of the Spearman correlation matrix of mutant counts by sample. Samples cluster according to time point and also by treatment (YPD *vs.* YPGal) and biological replicate.

Our results provide information directly relevant to designing future high-throughput quantitative DNA sequencing experiments of pooled mutants. For example, using an experimental design of four-fold biological replication and no technical replication, we showed that detection of mutants in the 4295 mutant yeast deletion collection with two-fold (or more) change between conditions can be achieved with 96% power at a 5% false discovery rate (FDR) using as few as 6 million reads per condition. This corresponds to a requirement of 1397 sequence reads per mutant per condition or 349 reads per biological replicate library. Using our experimental and analytical methods for Bar-seq analysis, it is possible to analyze the yeast deletion collection in up to 30 different conditions using a single 200-million read lane without sacrificing statistical power. Our findings should be informative for other methods of pooled mutant analysis such as Tn-seq.

## Materials and Methods

### Strains, media, and sampling procedures

We used a haploid prototrophic gene deletion collection constructed using the synthetic genetic array method (Tong *et al.* 2001). The library contains the identical gene deletion alleles as the standard yeast knockout collection (Winzeler *et al.* 1999), excluding gene deletions that result in auxotrophies. Gene deletion alleles are marked with the kanMX4 cassette conferring G418 resistance, which is flanked by a unique 5′ molecular barcode (the UPTAG) and a unique 3′ molecular barcode (the DNTAG). Each MAT**a** mutant contains a functional copy of the *URA3*, *LYS2*, *LEU2*, and *MET15* genes and the *can1*Δ::*STE2_pr_-SpHIS5*, *lyp1*Δ*0*, and *his3*Δ*1* alleles. We used standard YPD and YPGal media containing either 2% glucose or 2% galactose, respectively (Amberg *et al.* 2005).

After growth of individual mutants on YPD agar plates, all mutants were pooled to a final density of 1.5 × 10^9^ cells/ml. Each agar plate contained single colonies of individual genotypes and replicated colonies of the control *HO*Δ*0* strain. To define the replicated time zero (t0) samples, we obtained two independent samples of 0.5 ml (*i.e.*, 7.5 × 10^8^ cells) from the pooled library. We inoculated 5 μl from the pooled library (*i.e.*, 7.5 × 10^6^ cells) into four-fold replicated cultures of either 5 ml YPD or YPGal. Cells were grown for 24 hr (t24) to a final density of 3.3 × 10^8^ cells/ml in both conditions. We removed 2 ml (*i.e.*, 6.6 × 10^8^ cells) samples from each of the four YPD cultures and four YPGal cultures and purified genomic DNA using Qiagen Genomic-Tip 100 columns.

### Library preparation and sequencing

We designed a two-step PCR protocol for efficient multiplexing of Bar-seq libraries. In the first PCR step, UPTAGs from a single sample were amplified with the primers *Illumina UPTAG Index* (5′-ACG CTC TTC CGA TCT NNNNN GTC CAC GAG GTC TCT-3′) and *Illumina UPkanMX* (CAA GCA GAA GAC GGC ATA CGA GAT GTC GAC CTG CAG CGT ACG-3′), and DNTAGs from the same sample were amplified with the primers *Illumina DNTAG Index* (5′-ACG CTC TTC CGA TCT NNNNN GTG TCG GTC TCG TAG-3′) and *IlluminaDNkanMX* (5′-CAA GCA GAA GAC GGC ATA CGA GAT ACG AGC TCG AAT TCA TCG-3′) in separate PCR reactions. Illumina UPTAG and Illumina DNTAG primers contain a 5-bp sequence (denoted as NNNNN in the primer sequence) that uniquely identifies the sample. We designed 120 unique sample indices that differed by at least two nucleotides. A complete list of primer sequences is provided in Supporting Information, Table S1. We normalized genomic DNA concentrations to 10 ng/μl and using 100 ng template amplified barcodes using the following PCR program: 2 min at 98° followed by 20 cycles of 10 sec at 98°, 10 sec at 50°, 10 sec at 72°, and a final extension step of 2 min at 72°. PCR products were confirmed on 2% agarose gels and purified using QIAquick PCR purification columns.

We quantified purified PCR products using a Qubit fluorimeter and combined 60 ng from each of the 20 different UPTAG libraries and, in a separate tube, 60 ng from each of the 20 different DNTAG libraries. The multiplexed UPTAG libraries were then amplified using the primers *P5* (5′-A ATG ATA CGG CGA CCA CCG AGA TCT ACA CTC TTT CCC TAC ACG ACG CTC TTC CGA TCT-3′) and *Illumina UPkanMX*, and the combined DNTAG libraries were amplified using the *P5* and *IlluminaDNkanMX* primers using the identical PCR program as the first step with 20 ng template. The 140-bp UPTAG and DNTAG libraries were purified using QIAquick PCR purification columns, quantified using a Qubit fluorometer, combined in equimolar amounts, and adjusted to a final concentration of 10 nM (*i.e.*, 0.924 ng/μl). In total, the sequencing library contained 20 UPTAG and 20 DNTAG libraries from 20 different samples (Table S2). The library was sequenced on a single lane of an Illumina HiSeq 2000 using standard methods, including the use of the standard Illumina sequencing primer (5′-ACA CTC TTT CCC TAC ACG ACG CTC TTC CGA TCT-3′). The qseq files for each of the 20 samples are available from the NCBI Short Read Archive with the accession number SRA101498.

### Read matching and statistical analysis

Sequence reads were matched to the yeast deletion collection barcodes reannotated by Smith *et al.* (2009). Inexact matching was performed by identifying barcode sequences that were within a Levenshtein distance of 2 from each read (Levenshtein 1966). Reads matching equally to multiple barcodes were discarded. Sample indices were similarly matched using a maximum Levenshtein distance of 1. The final matrix of counts matching the UPTAG and DNTAG of each of the 20 samples is provided as Table S3. A set of 359 outliers was identified that had fewer than 100 total reads across all 20 samples (Figure S1). These low-count matches were likely due to sequencing error and were removed. In addition, our pooled yeast gene deletion library included a highly abundant strain (the HO gene deletion mutant, which was present on each of the 96-well plates containing individual mutants before pooling). The HO deletion mutant represented 19% of all reads and was removed before computational analyses, leaving a total of 139.8 million reads mapped to 4295 mutants.

Eigen *R*^2^ was used to determine the percent of variance explained by the different factors in our experimental design for the t_24_ samples (Chen and Storey 2008). Barcode counts were normalized using the TMM method (Robinson and Oshlack 2010) after adding 1 to each value and then were log-transformed, to avoid including differences in per library read depth as a source of variation. The bottom 10% of mutants was filtered out because lower counts have a disproportionate effect on the technical variation. Eigen *R*^2^ was used to compute the percent of variance explained by the treatment factor $\left(R_{T}^{2}\right)$ and the biological replicate factor $\left(R_{B}^{2}\right)$. Because the treatment factor is contained within the biological factor, we report the biological percent of variation as $R_{B}^{2}-R_{T}^{2}$, and the technical variation as $1-R_{B}^{2}-R_{T}^{2}$.

For differential abundance analysis, we first summed UPTAGs and DNTAGs for technical replicates within each biological replicate. The edgeR package (version 3.2.4) was used to perform dispersion estimation and to perform an exact negative binomial test to calculate a p-value and log-fold change for each mutant using the exactTest function using the default parameters (Robinson and Mccarthy 2010). The qvalue package was used to compute q-values (Storey and Tibshirani 2003).

Gene set enrichment analysis was performed using the Biological Process ontology from SGD. Gene sets that had fewer than four genes among the detected deletions were discarded in advance. We used the Wilcoxon rank-sum test to compare the distribution of the estimated log-fold changes within each gene set to those outside of the set (Gresham *et al.* 2011). We used the qvalue package to set a 5% FDR threshold, above which gene sets were declared significantly enriched.

### Read subsampling

Separate subsamplings were performed for each combination of replicates in each design. This requires one combination for the full 2 treatments × 4 biological replicates × 3 technical replicates design, two combinations for the 2 × 4 × 1 design, $\left(\begin{array}{c} 4\\ 3 \end{array}\right)=4$ combinations for the 2 × 3 × 2 design, $\left(\begin{array}{c} 4\\ 3 \end{array}\right)\times 2=8$ combinations for the 2 × 3 × 2 design, $\left(\begin{array}{c} 4\\ 2 \end{array}\right)=6$ combinations for the 2 ×2 × 2 design, and $\left(\begin{array}{c} 4\\ 2 \end{array}\right)\times 2=12$ combinations for the 2 × 2 × 1 design. For each combination, we performed subsampling over a sequence of 400 evenly spaced fractions of reads corresponding to 0.25%, 0.50%, …, 99.75%, and 100%.

For each fraction *p*, a subsampled count matrix *S* was generated based on the full experiment matrix as *S_i_*_,_*_j_* ∼Binom(*X_i_*_,_*_j_*, *p*). This is equivalent to choosing a random sample of the sequenced reads and then mapping them. The same analysis steps used for the full data set were used to analyze each subsample and the same metrics were applied to assess the results as used for the full experiment.

As the results for each experimental design depend on which of the replicates was chosen for subsampling the results were smoothed for each experimental design using a natural cubic spline with 20 degrees of freedom for estimates of the power, accuracy and FDR. For estimates of the informativeness of each experimental design, we used 15 degrees of freedom because the number of significant gene sets identified in each subsample showed greater variance than the other three metrics.

## Results

### Experimental results

We aimed to dissect the sources of variation in pooled mutant screens and to determine the appropriate analytical framework and experimental design that maximizes the value of the assay while minimizing cost and resources. All pooled genetic screens using mixtures of mutants require generation of a library of mutants, experimental treatment of the pooled mutants, and identification and quantification of DNA sequences that uniquely identify each mutant using high-throughput DNA sequencing. We designed an experiment to compare growth of haploid yeast nonessential gene deletion mutants in two different carbon sources, glucose (YPD) and galactose (YPGal), using Bar-seq analysis of the molecular barcodes that uniquely identify each mutant. To address the goals of our study, we prepared four biological replicates grown for 24 hr in each condition and two technical replicates (*i.e.*, independent sequencing library preparation of the same DNA sample) of each biological replicate (Figure 1A and Table S2). We also obtained two independent samples from the unselected library (time point 0) from which we prepared technical replicates.

To generate libraries for sequencing with an Illumina HiSeq, we designed a simple two-stage PCR protocol (see *Materials and Methods*). Each gene deletion is marked by two different molecular barcodes: one 5′ (the UPTAG) and one 3′ (the DNTAG) of the drug resistance cassette. To multiplex sequencing of different Bar-seq libraries, we developed a PCR-based method for library preparation that incorporates a unique sequence index for each library (see *Materials and Methods*). We sequenced 40 libraries (20 UPTAG and 20 DNTAG) from 20 samples in a single lane of an Illumina HiSeq 2000. We obtained 185.2 million reads that passed quality filters and matched them to the molecular barcodes by identifying sequences within a Levenshtein edit distance of 2, which resulted in mapping 93.3% of reads. Using a Levenshtein distance cutoff of 0 (*i.e.*, an exact match) or 1 results in successful mapping of 62.6% and 84.6% of the reads, respectively.

For the majority of mutants, the number of reads per barcode across all experiments follows an approximately log-normal distribution and ranges between 1000 and 100,000 (Figure S1). Low-count outliers that likely resulted from sequencing errors were removed (*Materials and Methods*). We found that UPTAGs and DNTAGs for each mutant had similar counts in the majority of samples, with 2574 mutants within a two-fold difference of each other (Figure S2). However, many mutants had highly divergent counts: 1264 had more than a 10-fold difference and 1052 had more than a 100-fold difference. These discrepancies were likely attributable to one of the barcodes being lost because of sequencing error in either the barcode or the PCR priming site.

Correlation analysis of barcode counts showed that the lowest correlations were between mutant abundance in the unselected library (t_0_) and mutant abundance after 24 hr of growth in either glucose-containing or galactose-containing media, indicating that differences in cell growth rates results in substantial changes in the relative abundance of mutants (Figure 1B). Growth in YPD yields higher correlation with the t_0_ sample than growth in YPGal, indicating that growth in galactose led to a greater shift in the relative abundance of mutants than did growth in glucose. To identify differential effects of mutants during growth in glucose and galactose, we restricted our analysis to the t_24_ samples. We used eigen *R*^2^ (Chen and Storey 2008) to partition the variance among these samples and found that 63.5% of the variance was explained by the treatment, 20.3% was explained by biological variation, and 16.1% was explained by technical variation (*Materials and Methods* ). The apportionment of variance was consistent across a wide range of percentile thresholds and using a variety of normalization methods (Figure S3).

### Computational analysis of differential mutant abundance

The goal of pooled mutant screens is to identify mutants that exhibit differences in abundance as a result of a defined treatment. The appropriate statistical methods depend on the nature of the data, which in the case of quantitative DNA sequencing of molecular barcodes are discrete count data. As we observed in the work of Gresham *et al.* (2011), the data are best described by an overdispersed Poisson distribution (*i.e.*, the variance of biological replicates is greater than the mean) (Figure S4). The problem of comparing count data between samples with different read depths while assuming overdispersed Poisson variation is related to that presented by differential expression analysis of RNA-seq data, for which a negative binomial test is used. In addition to the fact that Bar-seq data present some characteristics problematic for *t* tests (*i.e.*, lack of normality and a strong mean–variance relationship), there is important motivation for utilizing models specifically designed for count data. For example, consider two mutants in two different conditions in which the data of one are simply 1000× the other in read depth (*e.g.*, counts 8 and 9 *vs.* 13 and 14 for mutant *A* and 8000 and 9000 *vs.* 13,000 and 14,000 for mutant *B*). Whereas a *t* test results in the same p-value for both mutants, a negative binomial model directly takes into account the difference in read depth, resulting in drastically different p-values. Because the difference between the mutants with the lowest total number of reads to the highest number of reads is ∼2600-fold in our experiment (Figure S1), this is a valid issue. Therefore, we used a negative binomial model to test for mutants that were differentially abundant as a result of the treatment.

We utilized the edgeR software package (Robinson and McCarthy 2010), which has an efficient implementation of the negative binomial test that accounts for differing read depth and uses shrinkage to help estimate dispersion parameters. We observed that dispersion estimates underwent considerable shrinkage even when four biological replicates were used (Figure S4). We found RNA-seq analysis methods that also fit a negative binomial model, such as that implemented in DESeq (Anders and Huber 2010), produced qualitatively comparable results (Figure S5). Alternative methods, including DEGSeq (Wang *et al.* 2010) and Myrna (Langmead *et al.* 2010), make overdispersion assumptions less consistent with our data, whereas other methods, including Cuffdiff, use an implementation specific to RNA-seq (Trapnell *et al.* 2013).

Previous studies have used measurements of the UPTAG and DNTAG for each deletion mutant in different ways, including selection of the barcode for each mutant with the highest count (Smith *et al.* 2010) and independent analysis of each barcode (Gresham *et al.* 2011). Because the UPTAG and DNTAG were measurements of the same mutant, summing the counts within each sample provided a means of combining the information from both barcodes while remaining robust to cases in which one barcode was lost. Furthermore, with count data, summing across technical replicates provided a superior method for minimizing technical variation compared with calculating an average value. Therefore, we summed UPTAGs and DNTAGs for each mutant over technical replicates, such that each condition had four biological replicates, and applied tests using a negative binomial model to identify mutants that were significantly different in abundance in YPGal compared with YPD after 24 hr of growth. The 16 samples comprising this dataset included a total of 112 million reads.

**Figure 2:**
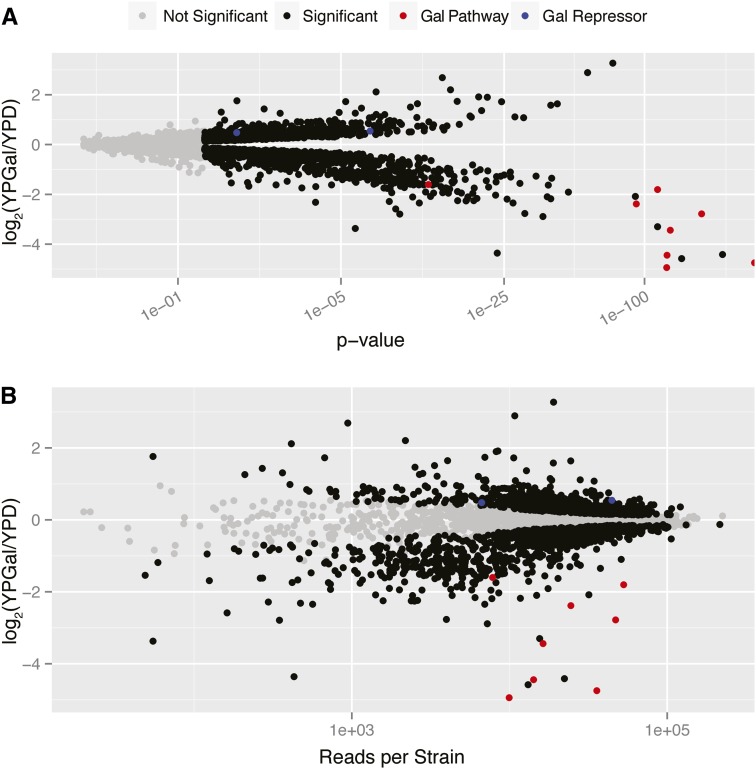
Bar-seq quantifies mutant effects across a range of sequence read depths. (A) Volcano plot showing the relationship between the p-value (log-scale) and log-fold change. Genes known to be involved in activation or repression of the galactose utilization pathway are highlighted. The p-value of the rightmost red point is computationally indistinguishable from 0. (B) Plot of reads per mutant in the entire experiment compared with the estimated fold change after treatment.

Analysis of our dataset identified 2036 mutants that were differentially abundant between the two conditions at 5% FDR. The effect sizes of individual gene deletions were widely distributed (Table S4 and Figure 2A). Notably, the gene deletion mutants for 8 of the 11 genes required for galactose metabolism (Timson 2007) were significantly decreased in abundance in YPGal and mutants deleted for two genes known to repress galactose metabolism were significantly increased in abundance in YPGal (Figure 2A). Gene set enrichment analysis using a Wilcoxon rank-sum test found 192 enriched gene sets at FDR of 5%, and the top sets were related to respiration and mitochondrial processes, consistent with the increased importance of respirative metabolism when yeast cells grow in galactose (Table S5). Mutants identified as significantly differing in abundance between YPGal and YPD were identified across a range of sequence read depths, although smaller effect sizes tended to be called statistically significant as read depth increased (Figure 2B). The ability to detect significant changes in mutant abundance was not greatly affected when total read counts were more than 1000, and two-fold differences were still detected as statistically significant with total read depths as low as 100. These observations suggest that we oversampled in our study and that similar results would be obtained with approximately an order of magnitude fewer reads.

### Effect of experimental design on statistical results

We aimed to identify the experimental design features that have the greatest effect on the results of a Bar-seq experiment. In practice, the experimental considerations that are most easily controlled are the extent of biological and technical replication and the depth to which each library is sequenced. We computationally simulated variation in each of these experiment design parameters using random subsampling of sequence reads from our complete experiment (*Materials and Methods*). For the purpose of assessing results from these subsamples, we compared them to results obtained from analysis of the complete dataset, which we defined as the gold standard. The negative binomial model we fit requires at least two biological replicates. Therefore, to study the effect of biological replication, we simulated the use of experimental designs using three or two biological replicates while retaining two technical replicates for each sample. To study the effect of technical replicates, we simulated the use of experimental designs using one technical replicate for each of the biological replicates. For each simulated experimental design, we sampled a subset of the reads to simulate varying read depths. We considered four metrics that assess the quality of each simulated experimental dataset: statistical power; accuracy; informativeness; and FDR.

**Figure 3:**
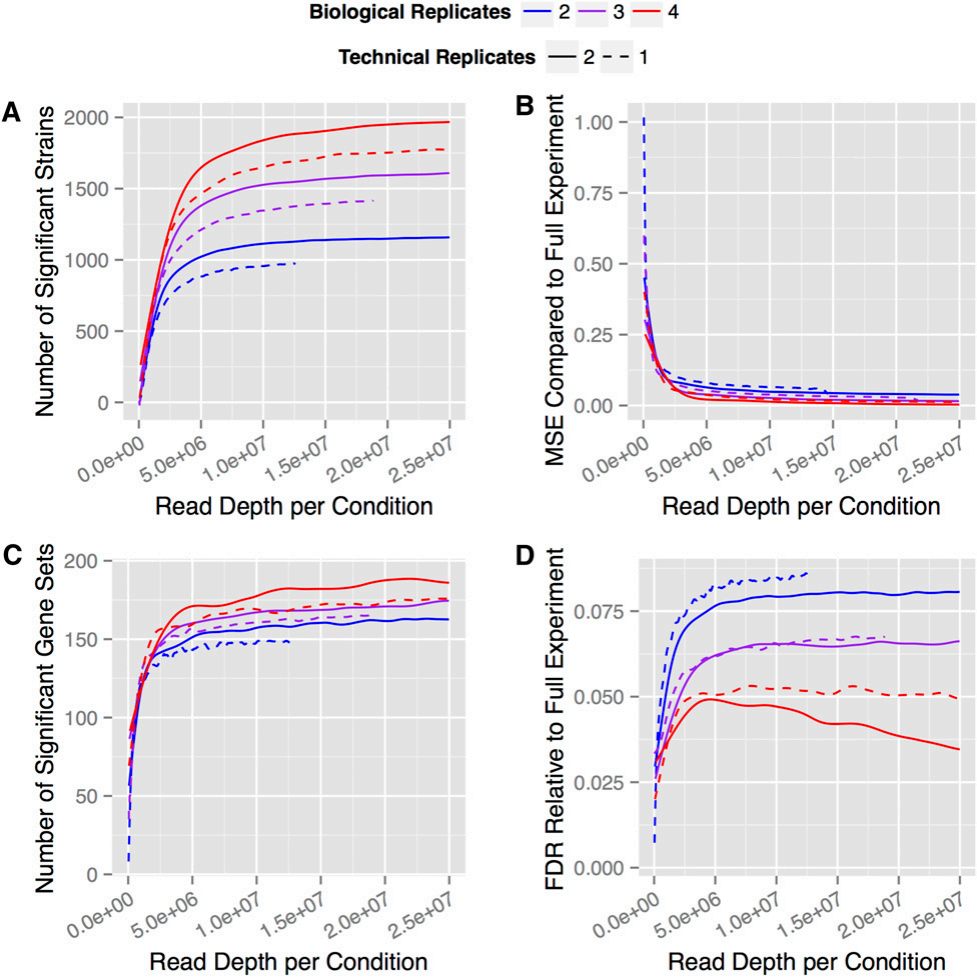
Simulation analysis of variation in experimental design. The effect of read depth on (A) the number of mutants found significant at FDR = 5%, (B) the mean squared error between the estimate of the log-fold change and the value for the full experiment, (C) the number of significant GO terms identified using a Wilcoxon rank-sum test at FDR = 5%, and (D) the percentage of significant genes that were not found to be significant in the full experiment. Curves are shown for the full experiment, 2 treatments × 4 biological replicates × 2 technical replicates, as well as for subsampled 2 × 3 × 2, 2 × 2 × 2 experimental designs (solid lines). Subsamplings were also performed to simulate each experimental design using a single technical replicate (dashed lines). Each curve was smoothed using a natural cubic spline.

We assessed the power of each experimental design for different sequence read depths by determining the number of mutants identified as differentially abundant at FDR of 5%. In all cases, the statistical power of each experimental design increased with read depth; however, it rapidly saturated (Figure 3A). Considering our full experimental design, it took just 1.7 million reads per condition to detect half of the significant mutants that were detected using the complete dataset and 75% were detected with 4.3 million reads per condition. Mutants that are most differentially abundant could be detected at very low read depths: the 13 most significant mutants identified using the complete dataset were all identified as significant even at the lowest depth tested, 140,000 mapped reads (*i.e.*, a 400-fold lower sequence read depth than the total), and were ranked among the 15 most significant mutants in all but the lowest read depth. Table S6 shows the effect size, significance, and rank of the seven most significant galactose-related genes at each level of subsampling, demonstrating that they remained highly significant even at very low read depths.

Reducing the number of biological replicates results in reduced statistical power for a given read depth. Using three biological replicates rather than four decreases the statistical power by approximately 16%, and using only two biological replicates decreases it by 38%. In practice, this effect is far more relevant than the read depth: 10 million mapped reads using two biological replicates achieves approximately the same power as 2 million total reads across four biological replicates, and the difference cannot be compensated by increasing sequence read depth. Technical replicates only marginally increase the power of the experimental design. This improvement is because pooling multiple replicates decreases the noise added by the library preparation and therefore decreases the within-treatment variation, analogous to previously studied strategies of pooling multiple replicates on a single microarray (Peng *et al.* 2003; Kendziorski *et al.* 2005).

Although the maximum power possible with each experimental design differs, it is interesting to note that the point at which statistical power begins to asymptote is very similar across experimental design, at approximately 6 million reads per condition (Figure 3A). This suggests that at this point, experimental noise attributable to the sequencing machine itself no longer decreases and additional variation is attributable to noise introduced by biological variability and library preparation. Statistical power varies within each subset of the designs depending on which replicates were selected (Figure S6), indicating that different replicates added different amounts of variance to the experiment, which cannot be predicted *a priori*.

The utility of an experimental design can also be assessed in terms of the accuracy with which effect sizes are estimated, as quantified by the mean square error, the informativeness of the analysis, as quantified by the number of significant gene sets identified by gene set enrichment analysis, or the FDR, as quantified by the proportion of genes found significant that are not significant in the full experiment. Assessments of the quality of each experimental design considering accuracy (Figure 3B), informativeness (Figure 3C), and FDR (Figure 3D) show that the greatest improvements are found with addition of biological replicates and that improvements beyond 6 million reads per condition are minimal, regardless of experimental design. Although there is some variation in the point at which each metric ultimately saturates, the points at which each metric begins to asymptote are highly concordant. Thus, beyond a surprisingly low threshold of 6 million reads per condition, additional sequencing depth provides little additional value.

**Figure 4:**
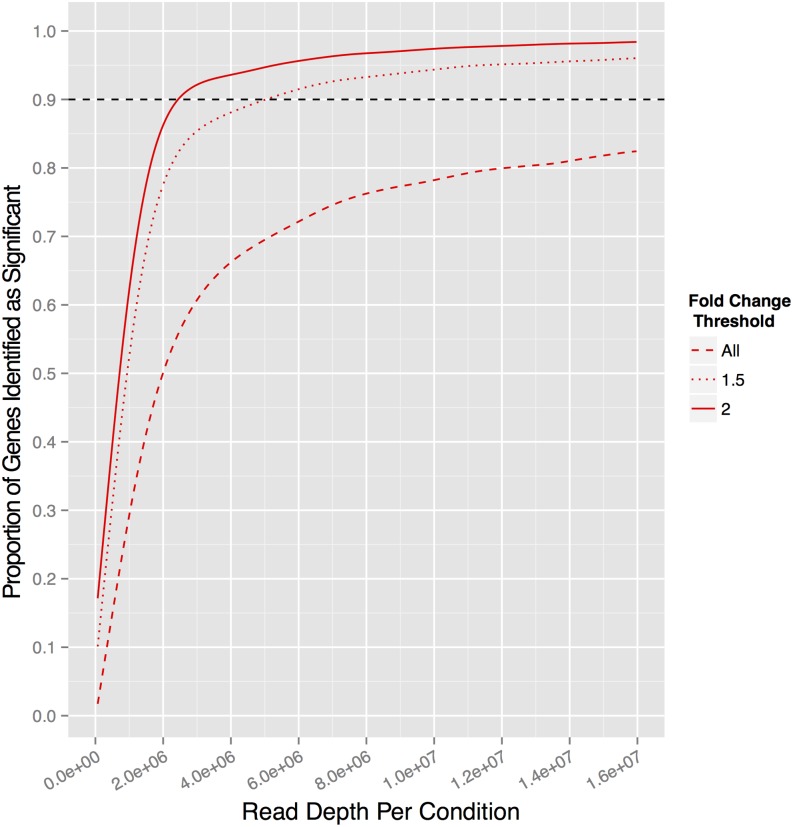
Statistical power varies with effect size and sequence read depth. The effect of read depth on the proportion of genes identified as significant (FDR = 5%) at different fold -change thresholds using 4 biological replicates × 1 technical replicate for each condition. The fold change for each mutant determined from the full 4 biological replicate × 2 technical replicate experiment is defined as the gold standard. The solid curve shows the proportion of genes found significant relative to the total experiment, whereas the dotted and dashed curves show the proportion of mutants that had at least a 1.5-fold or a two-fold change, respectively. The horizontal dashed line indicates the 90% power level.

Although pooled mutant screens enable simultaneous sensitive measurement of the effect of each mutant, they are frequently used as a means of identifying those mutants of greatest effect. We analyzed the statistical power of an experimental design using four biological replicates and no technical replication for different effect sizes (Figure 4). As few as 2.5 million sequence reads per condition (625,000 reads per sample) are sufficient to detect 90% of mutants that change more than two-fold in the full experiment. Increasing the read depth to 6 million reads per condition detects 96% of mutants that change more than two-fold, 91% of all mutants that change more than 1.5-fold and 72% of all mutants that are significant in the full experiment.

## Discussion

High-throughput quantitative DNA sequencing has resulted in rapid advances in a range of problems from the analysis of genome variation to the three-dimensional organization of genomes. The coupling of high-throughput quantitative sequencing with large-scale mutagenesis (either systematic or random) enables the pooled analysis of mutant phenotypes with broad applications, including the study of gene function, drug targets, and genetic interactions. Here, we have studied one realization of pooled mutant analysis, Bar-seq, with the goal of determining experimental designs and analytical methods that provide excellent levels of sensitivity, specificity, and efficiency.

We have shown that statistical models used for RNA-seq analysis are directly applicable to the analysis of Bar-seq data. Tools for RNA-seq analysis, such as those used here, are therefore readily adapted to Bar-seq analysis, providing estimates of effect sizes and statistical significance for each mutant. For Bar-seq analysis, UPTAGs and DNTAGs represent additive measurements of the same genotype and therefore should be summed for each sample. Similarly, technical replicates should be combined by addition of barcode counts.

Biological replication is essential for rigorous assessment of statistical significance. At least two biological replicates should always be performed to use the within-treatment variation for determining statistical significance. Some software packages have the option of guessing the dispersion in advance, but this is not recommended because an incorrect estimate would make subsequent tests for statistical significance either too conservative or too generous. Moreover, we have found that different experiments can contribute different amounts of variation. Therefore, we recommend performing at least four biological replicates to maximize statistical power and accuracy of effect size. The use of technical replicates of biological replicates results in marginal improvements and is likely unnecessary.

Importantly, we found that Bar-seq does not require a high read depth to accurately detect differential abundance of mutants and that additional reads add little to the results. In our study using nearly 60 million mapped reads per condition to analyze 4295 mutants, we demonstrated that the quality of our dataset was maintained with approximately 10-fold fewer reads. Our experimental method for Bar-seq includes 120 uniquely indexed adaptors (Table S2), meaning that on a 200-million read sequencing lane, one can analyze four biological replicates of 30 different conditions, resulting in approximately 6 million reads per condition. Based on our analysis, that read depth would be expected to identify 96% of genes with a two-fold change, 91% of mutants with a 1.5-fold change, and 72% of all mutants that would be detected with 10-times greater read depth and two technical replicates (Figure 4). These findings can be extended to other methods for pooled genetic screens by noting that it corresponds to ∼1400 reads per genomic target per condition. Increasing sequence read depth beyond this value provides only an incremental increase. Thus, our analysis provides guidelines about the tradeoff between per-condition read depth and statistical power that can be used for the design of future experiments.

## Supplementary Material

Supporting Information
