# Supplementary material for: Design and Analysis of Bar-seq Experiments
Source: G3 (Bethesda). 2013 Nov 5;4(1):11–8. doi: 10.1534/g3.113.008565 (PMC3887526; doi:10.1534/g3.113.008565)
Supplement: Supporting Information [file supp_4_1_11__index.html]

Design and Analysis of Bar-seq Experiments — Supporting Information 

# Design and Analysis of Bar-seq Experiments

## Supporting Information for Robinson *et al.*, 2014

**Files in this Data Supplement:**

- Supporting Information - Figures S1-S6, File S1, and Tables S1-S6 (PDF, 984 KB)
- Figure S1 - Distribution of the number of reads for all identified mutants. (PDF, 465 KB)
- Figure S2 - Comparison of UPTAG and DNTAG counts for each mutant. (PDF, 495 KB)
- Figure S3 - The percent of variance explained by treatment and biological and technical replication as determined by eigen-*R*2. (PDF, 480 KB)
- Figure S4 - Comparison of mean barcode count with the associated variance. (PDF, 523 KB)
- Figure S5 - P-values for the YPD/YPGal comparison for each mutant, calculated using the negative binomial models with edgeR and DESeq. (PDF, 496 KB)
- Figure S6 - The number of significant mutants at different read depths for different subsets of subsampling experiments. (PDF, 502 KB)
- Table S1 - 120 UPTAG and DNTAG indexed primer sequences for multiplexed Bar-seq analysis of the yeast deletion collection (.xlsx, 46 KB)
- Table S2 - The UPTAG and DOWNTAG primer and index used for each of the 20 samples analyzed in the current study (.xlsx, 47 KB)
- Table S3 - The matrix of raw read counts that matched to each tag in each replicate (.xlsx, 1 MB)
- Table S4 - The p-value and q-value for the test for differential abundance using both DESeq and edgeR for each mutant (.xlsx, 514 KB)
- Table S5 - The p-values for gene set enrichment analysis using the Wilcoxon rank-sum test on the estimated log2 fold changes (.xlsx, 257 KB)
- Table S6 - The estimated log fold change, q-value, and significance rank for the 7 most significant GAL genes at each of the 400 levels of read subsampling (.xlsx, 146 KB)
- File S1 - Code for reproducing analysis and text of manuscript (.zip, 2 MB)
