# Supplementary material for: Design and Analysis of Bar-seq Experiments
Source: G3 (Bethesda). 2013 Nov 5;4(1):11–8. doi: 10.1534/g3.113.008565 (PMC3887526; doi:10.1534/g3.113.008565)
Supplement: Supporting Information [file supp_g3.113.008565_008565SI.pdf]

G3 Genes | Genomes | Genetics

Design and Analysis of Bar-seq Experiments

David G. Robinson[1], Wei Chen[2], John D. Storey[1,4], and David Gresham[3,4]

[1]Lewis-Sigler Institute for Integrative Genomics, Princeton University, Princeton.
[2]Berlin Institute for Medical Systems Biology, Max-Delbruck-Center for Molecular Medicine, Berlin, Germany.
[3]Center for Genomics and Systems Biology, Department of Biology, New York University, New York.
[4]correspondence: dgresham@nyu.edu and jstorey@princeton

DOI: 10.1534/g3.113.008565

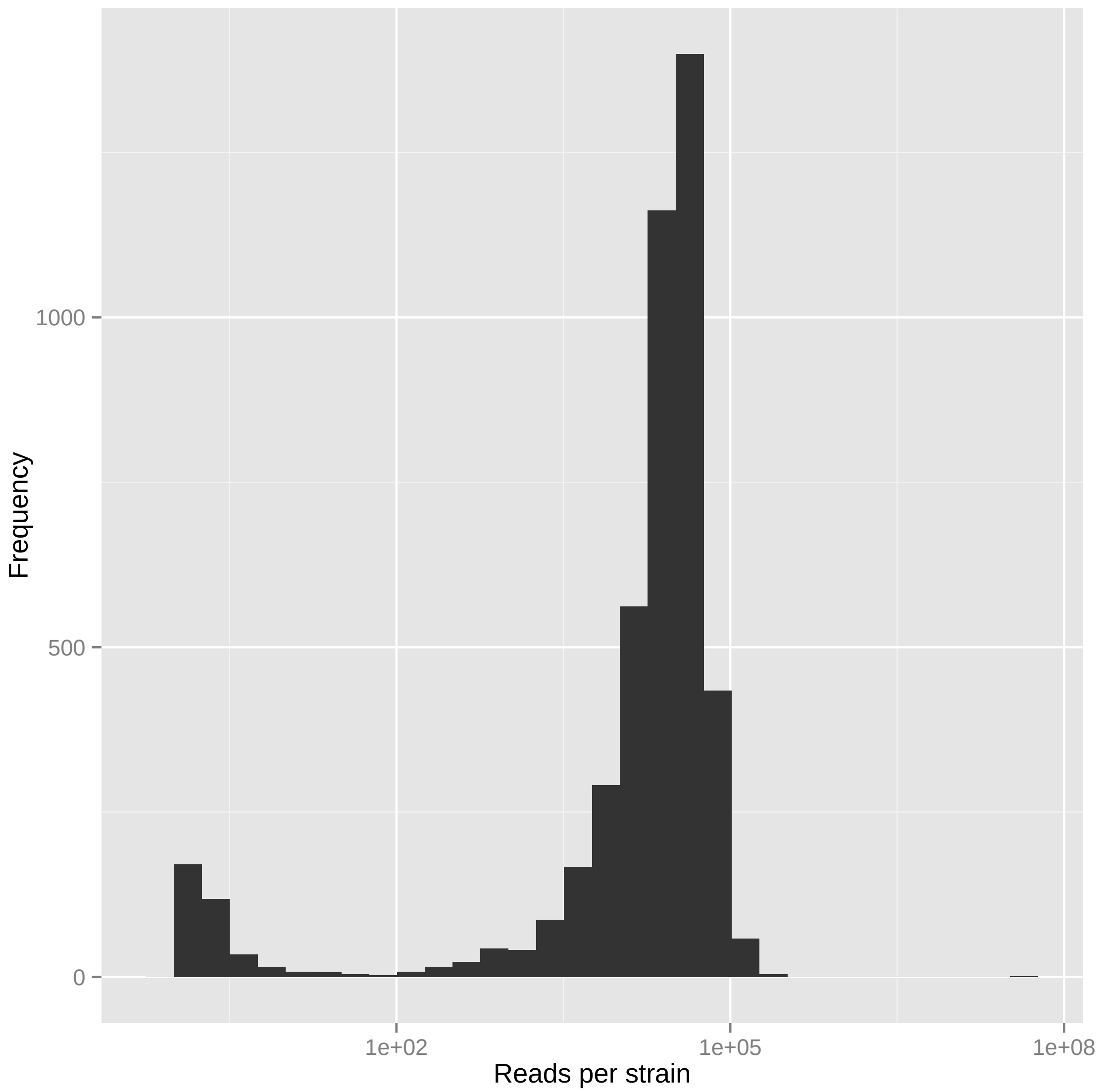

Figure S1: **Distribution of the number of reads for all identified mutants.** Most mutants follow an approximately log-normal distribution in terms of their abundance, with an additional group of mutants that had fewer than 100 counts across all 20 samples, probably due to sequencing error.

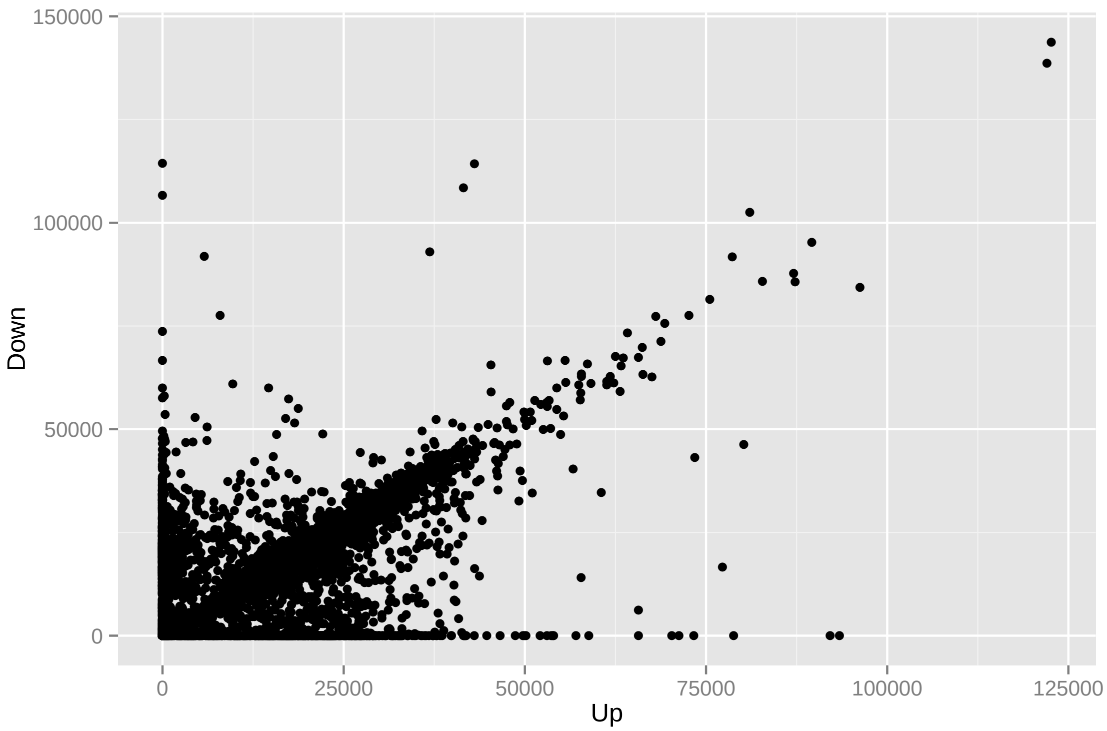

Figure S2: **Comparison of UPTAG and DNTAG counts for each mutant.** While the counts were closely correlated for many mutants, a large proportion of mutants had unusually low counts for one barcode, with some missing either an UPTAG or DNTAG entirely, probably due to a mutation in the barcode or the primer.

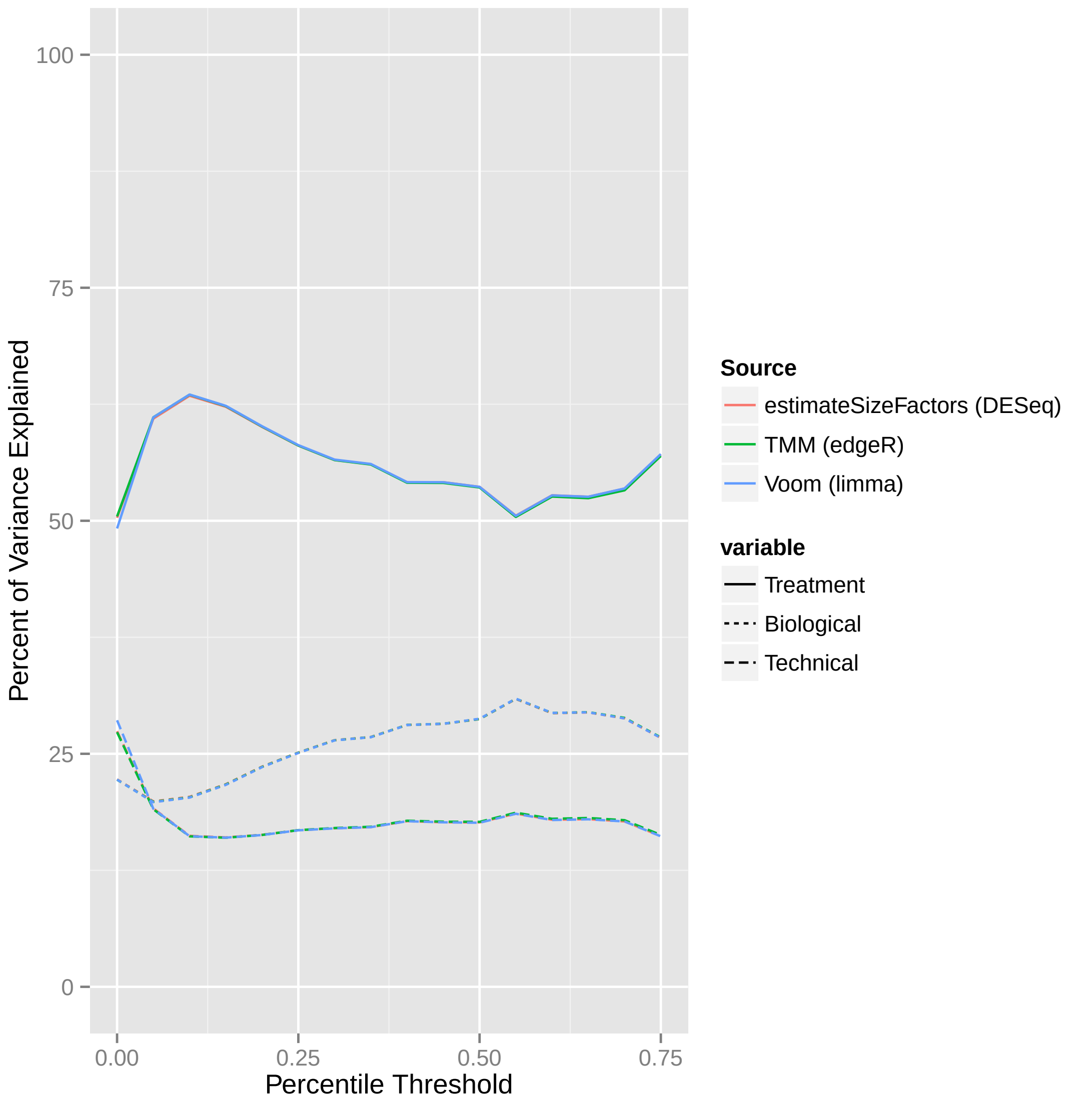

Figure S3: **The percent of variance explained by treatment and biological and technical replication as determined by eigen-R^2.** The results are qualitatively identical regardless of the normalization method and the percentile threshold for the minimum number of required reads for inclusion of a mutant.

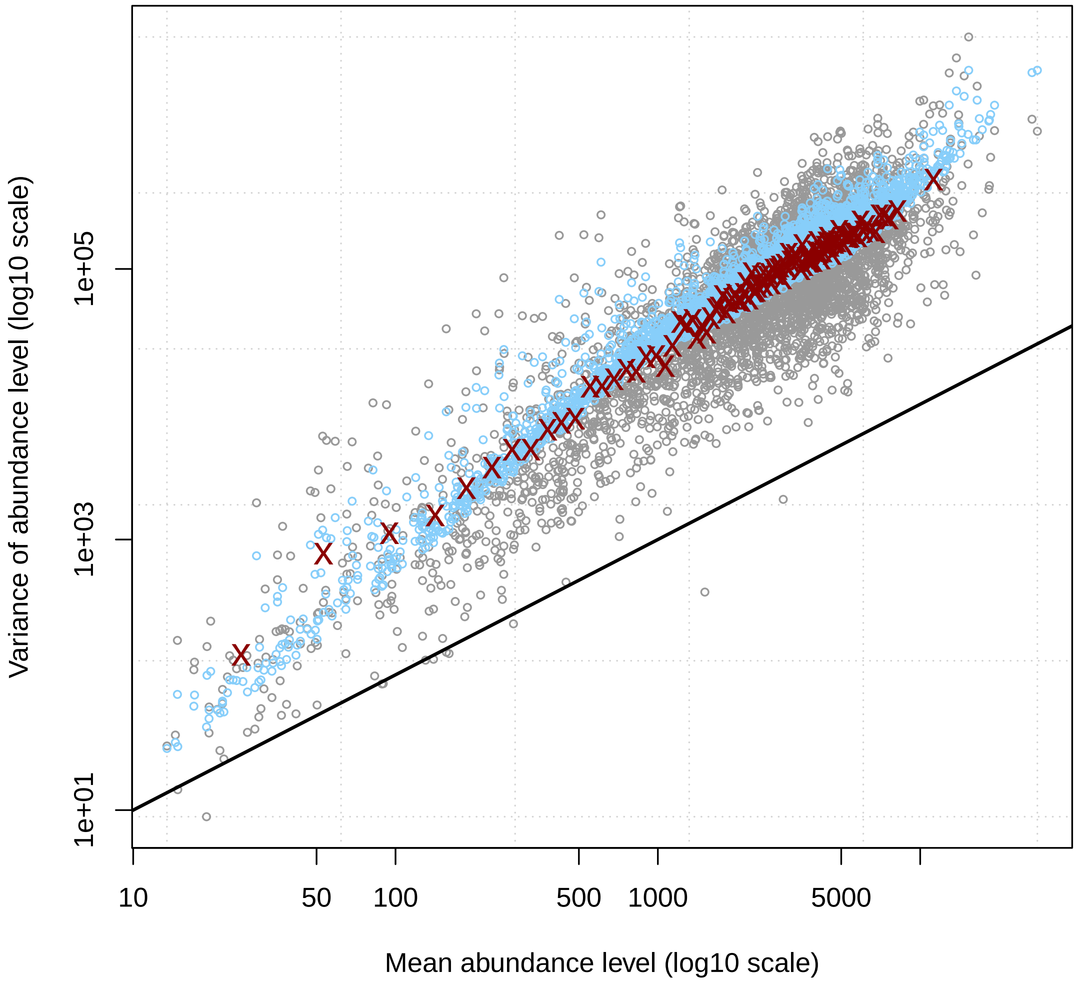

Figure S4: **Comparison of mean barcode count with the associated variance.** The x-axis shows the mean barcode count of each mutant, the y-axis shows the pooled variance within each experimental condition after accounting for read depth. Grey points are the raw measurements for each mutant, the red X's are the average variance in each bin, and the blue points are the estimated variance of each mutant after dispersion shrinkage has been performed. Variance tends to be substantially greater than the mean suggesting that a overdispersed Poisson or negative binomial model is appropriate.

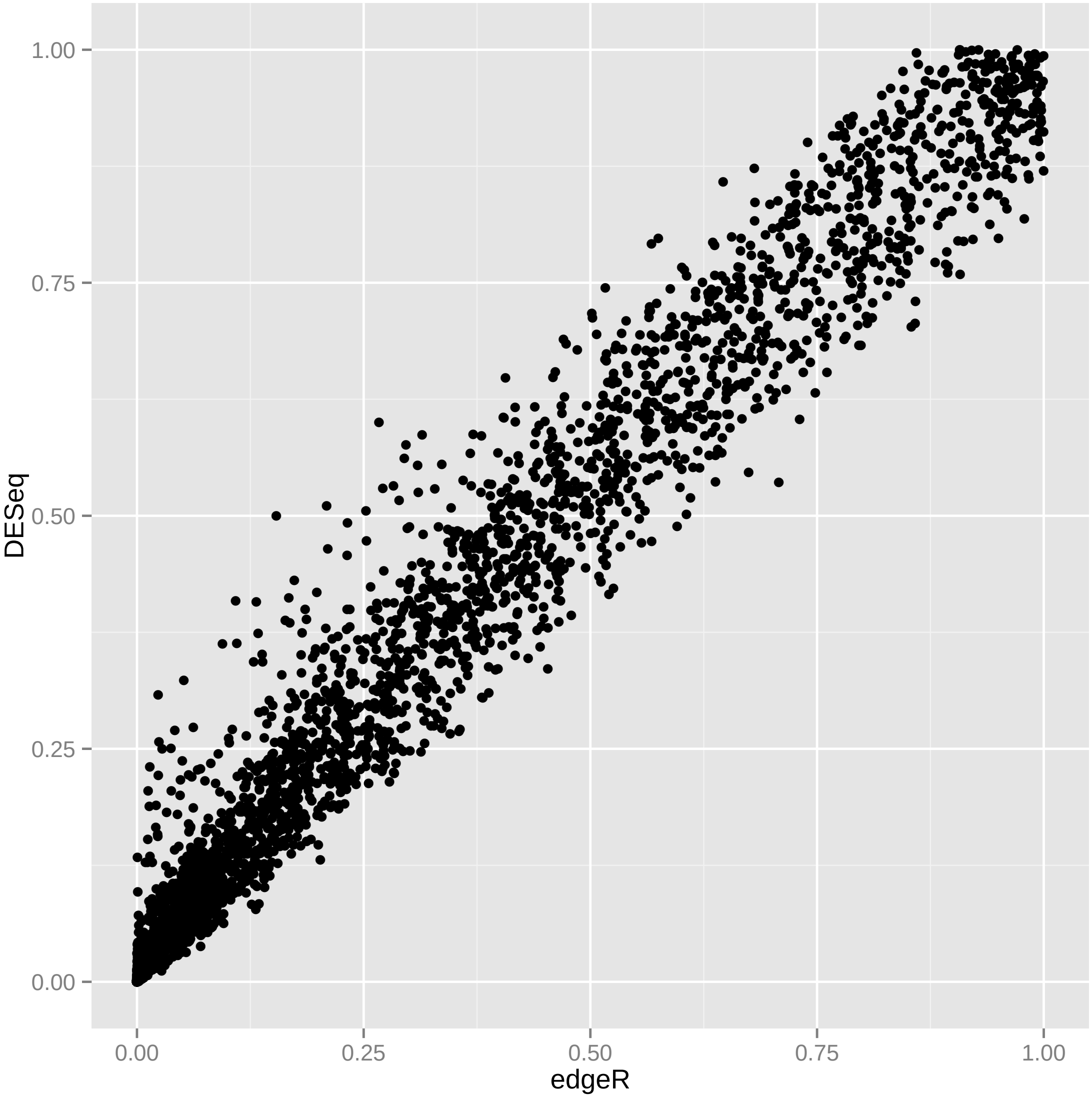

Figure S5: **P-values for the YPD/YPGal comparison for each mutant, calculated using the negative binomial models with `edgeR` and `DESeq`.** The methods show a Spearman correlation of 0.99, indicating only slight differences in their approach.

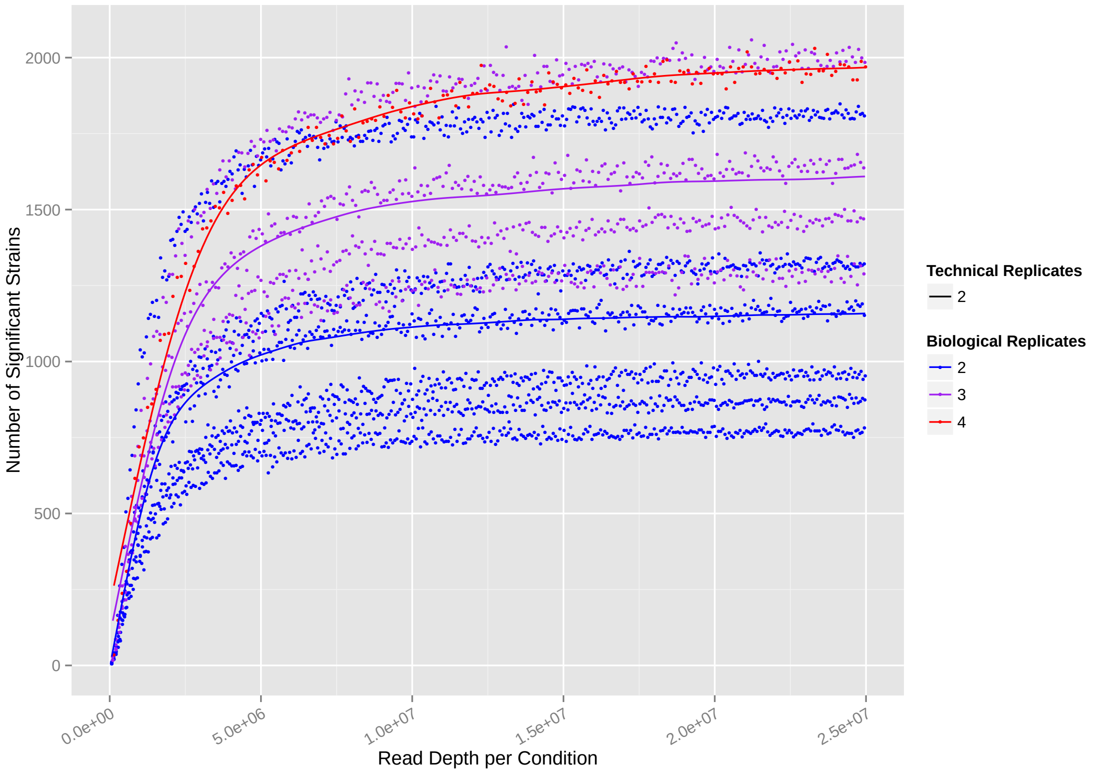

Figure S6: **The number of significant mutants at different read depths for different subsets of subsampling experiments**. A spline is fit to the results for each of comparison.

File S1

Code for reproducing analysis and paper

Available for download as a .zip file at http://www.g3journal.org/lookup/suppl/doi:10.1534/g3.113.008565/-/DC1.

Tables S1-S6
Available for download at http://www.g3journal.org/lookup/suppl/doi:10.1534/g3.113.008565/-/DC1.

Table S1 120 UPTAG and DNTAG indexed primer sequences for multiplexed Bar- seq analysis of the yeast deletion collection.

Table S2 The UPTAG and DOWNTAG primer and index used for each of the 20 samples analyzed in the current study.

Table S3 The matrix of raw read counts that matched to each tag in each replicate. The first three columns give the systematic and gene name of the deletion and an indication as to whether the mutant was among the 4295 included in the analysis.

Table S4 The p-value and q-value for the test for differential abundance using both DESeq and edgeR for each mutant. Also shown are the estimated $\log_2$ fold changes, the total number of reads matching the gene across both conditions, and the annotation of the biological process indicated in Figure 2.

Table S5 The p-values for gene set enrichment analysis using the Wilcoxon rank-sum test on the estimated $\log_2$ fold changes. The gene sets shown are those in the Biological Process ontology that had at least four genes in the set of analyzed deletions.

Table S6 The estimated log fold change, q-value, and significance rank for the 7 most significant GAL genes at each of the 400 levels of read subsampling.
