# Supplementary material for: Design and Analysis of Bar-seq Experiments
Source: G3 (Bethesda). 2013 Nov 5;4(1):11–8. doi: 10.1534/g3.113.008565 (PMC3887526; doi:10.1534/g3.113.008565)
Supplement: Supporting Information [file supp_g3.113.008565_FigureS1.pdf]

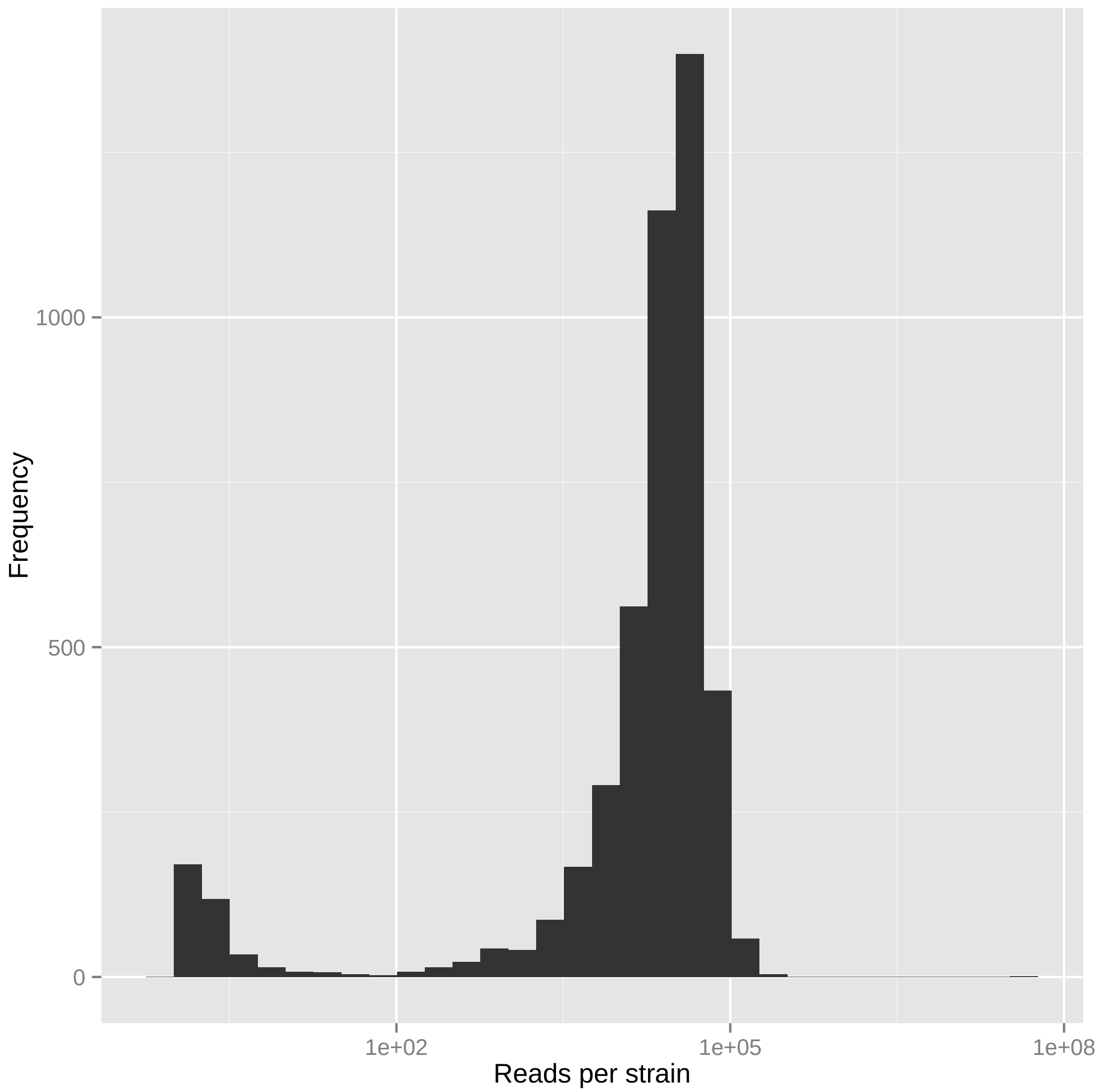

Figure S1: **Distribution of the number of reads for all identified mutants.** Most mutants follow an approximately log-normal distribution in terms of their abundance, with an additional group of mutants that had fewer than 100 counts across all 20 samples, probably due to sequencing error.
