# Supplementary material for: Design and Analysis of Bar-seq Experiments
Source: G3 (Bethesda). 2013 Nov 5;4(1):11–8. doi: 10.1534/g3.113.008565 (PMC3887526; doi:10.1534/g3.113.008565)
Supplement: Supporting Information [file supp_g3.113.008565_FigureS2.pdf]

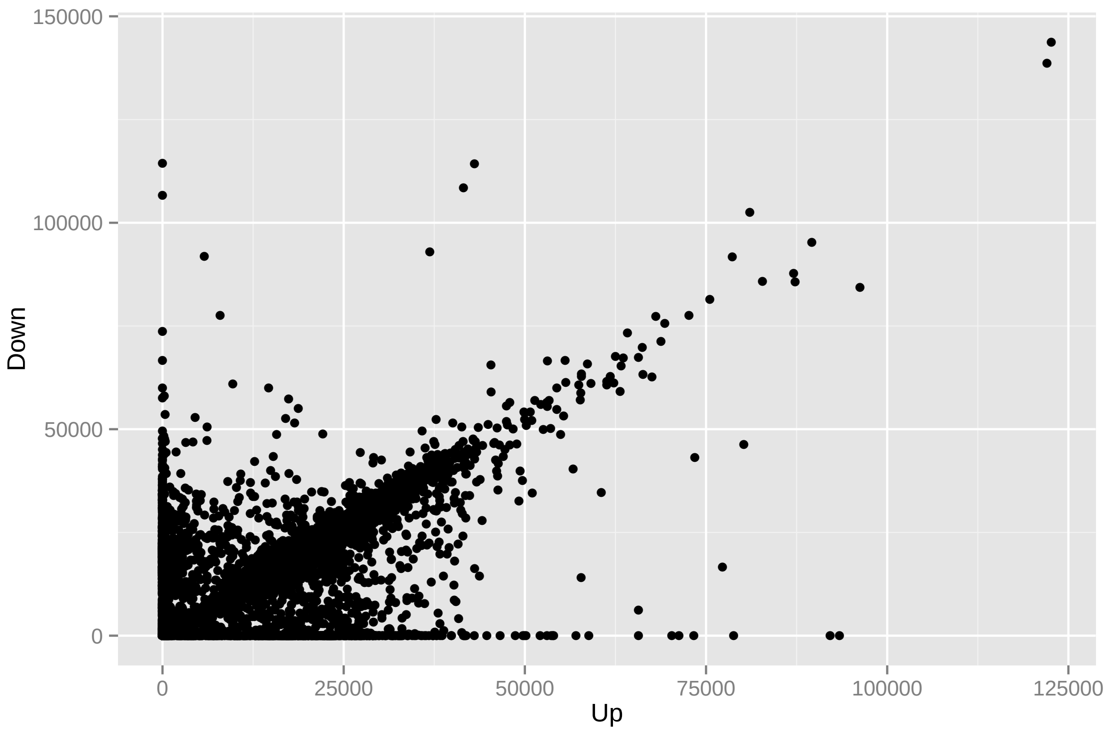


Figure S2: **Comparison of UPTAG and DNTAG counts for each mutant.** While the counts were closely correlated for many mutants, a large proportion of mutants had unusually low counts for one barcode, with some missing either an UPTAG or DNTAG entirely, probably due to a mutation in the barcode or the primer.
