# Supplementary material for: Design and Analysis of Bar-seq Experiments
Source: G3 (Bethesda). 2013 Nov 5;4(1):11–8. doi: 10.1534/g3.113.008565 (PMC3887526; doi:10.1534/g3.113.008565)
Supplement: Supporting Information [file supp_g3.113.008565_FigureS3.pdf]

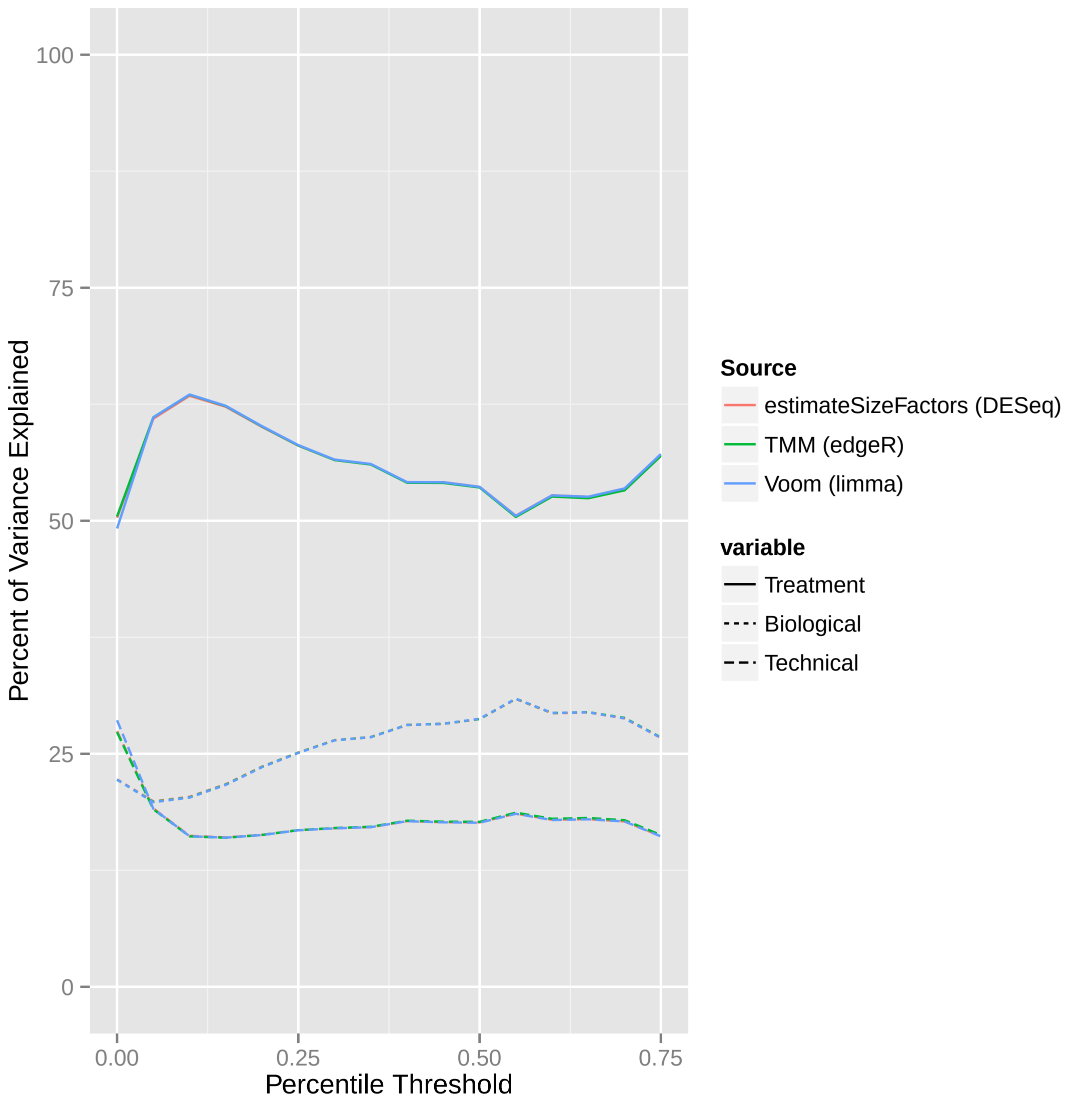

Figure S3: **The percent of variance explained by treatment and biological and technical replication as determined by eigen-R^2.** The results are qualitatively identical regardless of the normalization method and the percentile threshold for the minimum number of required reads for inclusion of a mutant.
