# Supplementary material for: Design and Analysis of Bar-seq Experiments
Source: G3 (Bethesda). 2013 Nov 5;4(1):11–8. doi: 10.1534/g3.113.008565 (PMC3887526; doi:10.1534/g3.113.008565)
Supplement: Supporting Information [file supp_g3.113.008565_FigureS4.pdf]

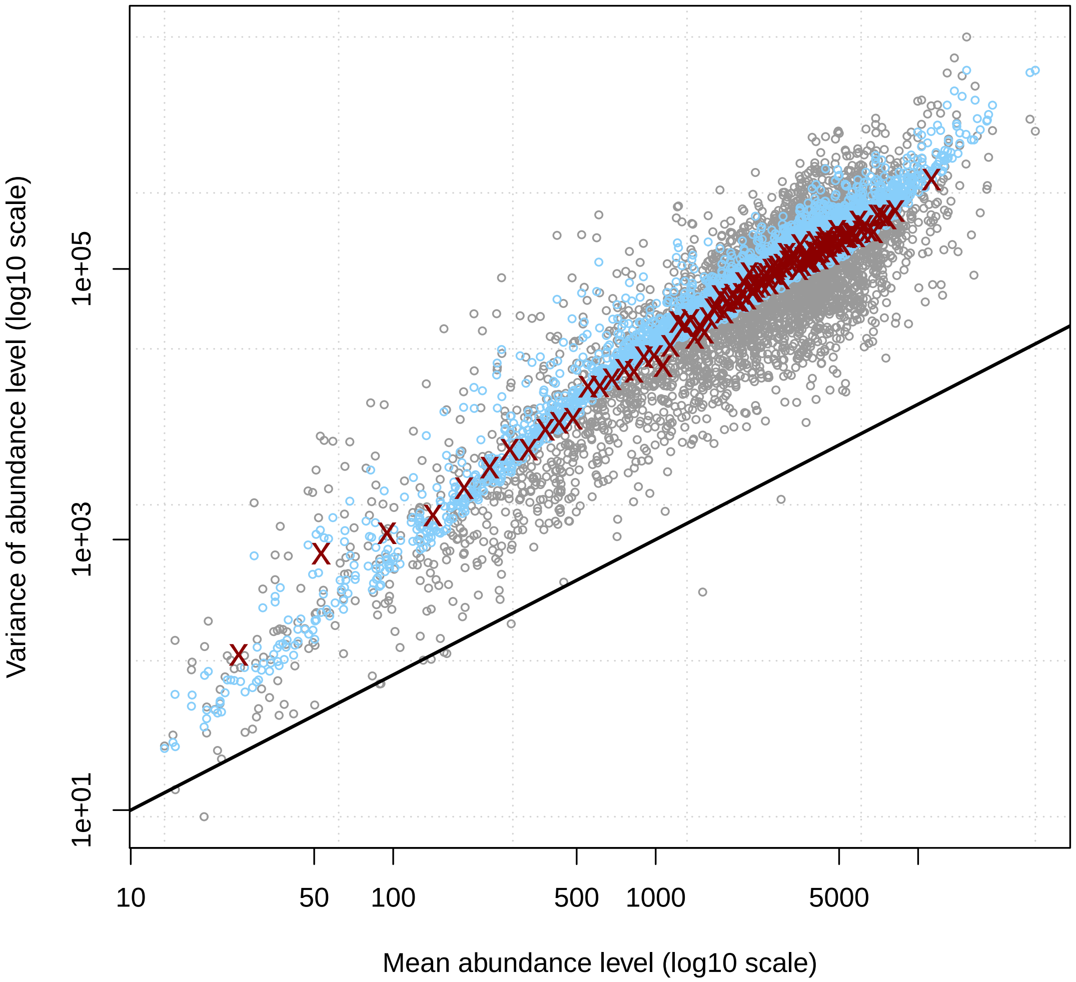

Figure S4: **Comparison of mean barcode count with the associated variance.** The x-axis shows the mean barcode count of each mutant, the y-axis shows the pooled variance within each experimental condition after accounting for read depth. Grey points are the raw measurements for each mutant, the red X's are the average variance in each bin, and the blue points are the estimated variance of each mutant after dispersion shrinkage has been performed. Variance tends to be substantially greater than the mean suggesting that a overdispersed Poisson or negative binomial model is appropriate.
