# Supplementary material for: Design and Analysis of Bar-seq Experiments
Source: G3 (Bethesda). 2013 Nov 5;4(1):11–8. doi: 10.1534/g3.113.008565 (PMC3887526; doi:10.1534/g3.113.008565)
Supplement: Supporting Information [file supp_g3.113.008565_FigureS5.pdf]

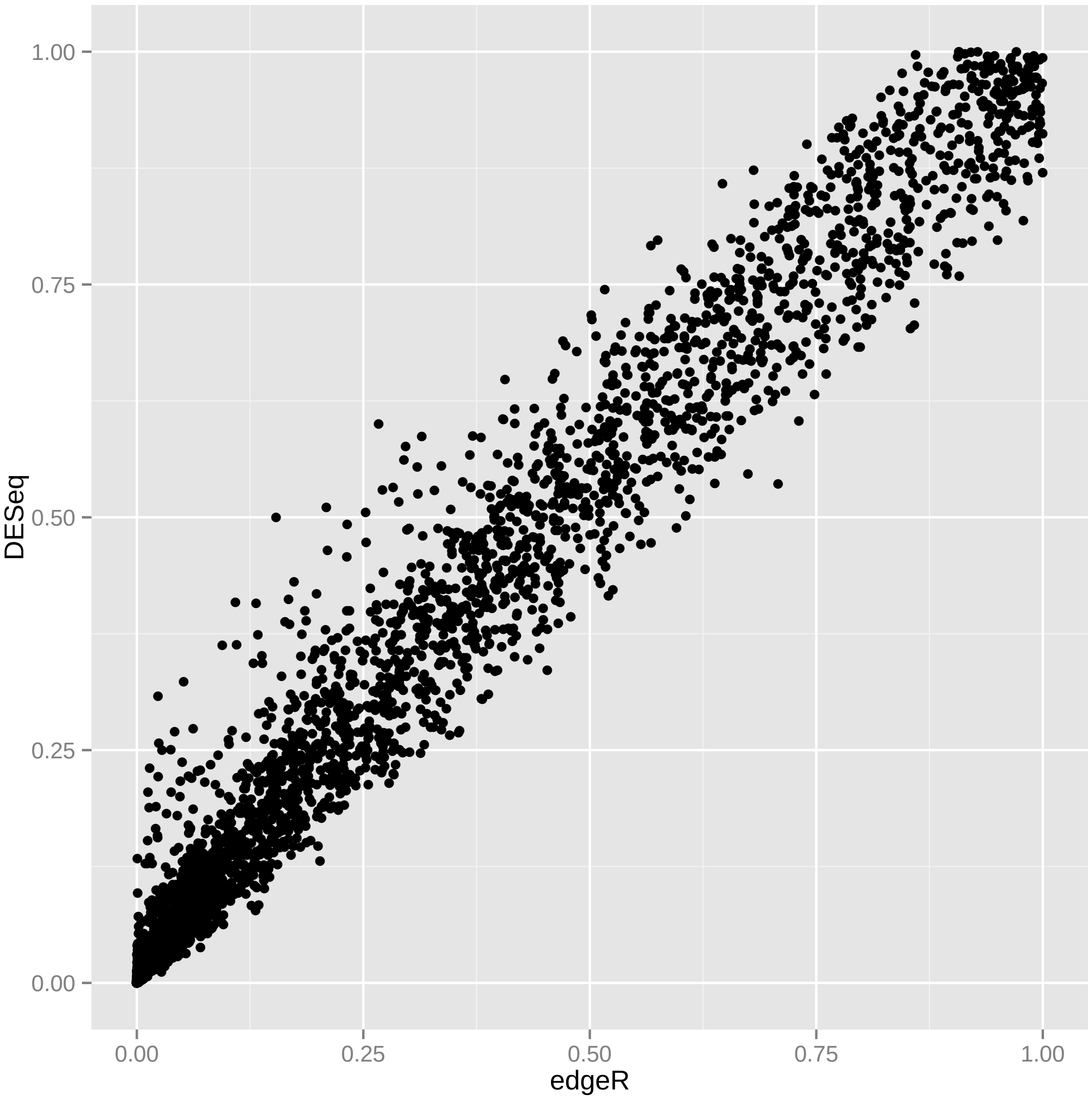

Figure S5: **P-values for the YPD/YPGal comparison for each mutant, calculated using the negative binomial models with `edgeR` and `DESeq`.** The methods show a Spearman correlation of 0.99, indicating only slight differences in their approach.
