# Supplementary material for: Design and Analysis of Bar-seq Experiments
Source: G3 (Bethesda). 2013 Nov 5;4(1):11–8. doi: 10.1534/g3.113.008565 (PMC3887526; doi:10.1534/g3.113.008565)
Supplement: Supporting Information [file supp_g3.113.008565_FigureS6.pdf]

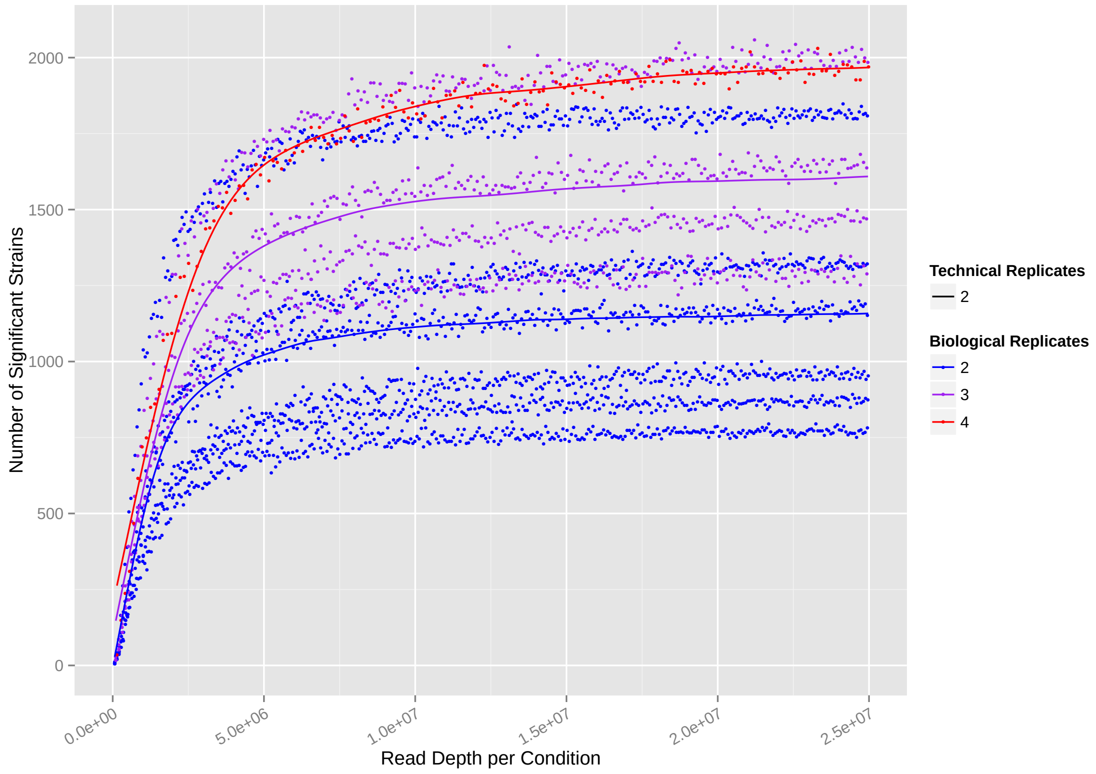

Figure S6: **The number of significant mutants at different read depths for different subsets of subsampling experiments**. A spline is fit to the results for each of comparison.
